# Enhancing research data infrastructure to address the opioid epidemic: the Opioid Overdose Network (O2-Net)

**DOI:** 10.1093/jamiaopen/ooac055

**Published:** 2022-06-30

**Authors:** Leslie A Lenert, Vivienne Zhu, Lindsey Jennings, Jenna L McCauley, Jihad S Obeid, Ralph Ward, Saeed Hassanpour, Lisa A Marsch, Michael Hogarth, Perry Shipman, Daniel R Harris, Jeffery C Talbert

**Affiliations:** Biomedical Informatics Center, Medical University of South Carolina, Charleston, South Carolina, USA; Biomedical Informatics Center, Medical University of South Carolina, Charleston, South Carolina, USA; Department of Emergency Medicine, Medical University of South Carolina, Charleston, South Carolina, USA; Department of Psychiatry, Medical University of South Carolina, Charleston, South Carolina, USA; Biomedical Informatics Center, Medical University of South Carolina, Charleston, South Carolina, USA; Department of Public Health Sciences, Medical University of South Carolina, Charleston, South Carolina, USA; Biomedical Data Science Department, Geisel School of Medicine, Dartmouth College, Lebanon, New Hampshire, USA; Center for Technology and Behavioral Health, Geisel School of Medicine, Dartmouth College, Lebanon, New Hampshire, USA; Department of Biomedical Informatics, University of California San Diego, San Diego, California, USA; Altman Clinical and Translational Research Institute, University of California San Diego, San Diego, California, USA; Institute for Biomedical Informatics, University of Kentucky, Lexington, Kentucky, USA; Institute for Biomedical Informatics, University of Kentucky, Lexington, Kentucky, USA

**Keywords:** opioid abuse, opioid overdose, clinical trials, e-phenotype, natural language processing, electronic health records systems

## Abstract

Opioid Overdose Network is an effort to generalize and adapt an existing research data network, the Accrual to Clinical Trials (ACT) Network, to support design of trials for survivors of opioid overdoses presenting to emergency departments (ED). Four institutions (Medical University of South Carolina [MUSC], Dartmouth Medical School [DMS], University of Kentucky [UK], and University of California San Diego [UCSD]) worked to adapt the ACT network. The approach that was taken to enhance the ACT network focused on 4 activities: cloning and extending the ACT infrastructure, developing an e-phenotype and corresponding registry, developing portable natural language processing tools to enhance data capture, and developing automated documentation templates to enhance extended data capture. Overall, initial results suggest that tailoring of existing multipurpose federated research networks to specific tasks is feasible; however, substantial efforts are required for coordination of the subnetwork and development of new tools for extension of available data. The initial output of the project was a new approach to decision support for the prescription of naloxone for home use in the ED, which is under further study within the network.

## INTRODUCTION

This paper describes work to adapt an existing nationwide clinical federated data network to better assess and conduct trials to combat the opioid overdose (OOD) epidemic. Opioid misuse and dependence continue to be a significant and growing cause of preventable morbidity and mortality in the United States.^1^ Over the last 10 years, the number of individuals presenting to emergency departments (EDs) as a result of intentional or unintentional opioid-related overdose (OD) has dramatically increased.^2^^,^^3^ Many of these individuals die or have a repeat OD presentation within a year^4^^,^^5^ and public health costs associated with opioid-related OD are high.^2^^,^^6^^,^^7^ The defining features of the opioid epidemic have changed over time. While prescription opioid misuse was initially a major contributing problem, increasing use of high potency nonprescription drugs, such as heroin, and illicit fentanyl has become the major cause of opioid-related ED presentations and mortality.^3^^,^^8^ Recent data suggest that a growing proportion of opioid-dependent individuals are initiating use with heroin (or illicit synthetic opioids), rather than prescribed opioids,^9^^,^^10^ contributing to public health experts’ beliefs that opioid-related OD may continue to increase over the next 1–5 years.^9^^,^^10^

Patients presenting with OOD to EDs represent an extraordinarily high-risk group for mortality (up to a 24-fold risk ratio),^11^ and thus are an important target for interventions to combat the opioid epidemic both from the social need and trial design perspective (higher event rates in a population can make the detection of efficacy easier). The ability to characterize these individuals could inform trials of multilevel, multisystem OD prevention efforts, as well as translational research efforts to connect opioid-dependent individuals with effective treatments.

Recognizing that accuracy and detail in preliminary data are essential when designing effective clinical and translational studies (research-driven, interactive access to data is preferable through tools such as i2b2^12^) we have undertaken foundational work to extend the Accrual to Clinical Trials (ACT^13^) network to create an interinstitutional research database and network focused on accelerating clinical and translational research for opioid-use disorder in the context of OOD. To develop a testbed for adapting the ACT network to opioid use disorder (OUD) and OOD scenarios, we worked with 3 network partners: the University of California San Diego (UCSD), the University of Kentucky (UK), and Dartmouth CTSA sites. The approach entailed (1) e-phenotype for case identification in the ED based on (electronic health record) EHR data, combined this (2) use of natural language processing (NLP) and artificial intelligence (AI) algorithms to abstract critical concepts, and (3) EHR tools for direct entry of data during clinical care in the ED to supplement data. Each approach was designed in the context of cross-institution collaboration, allowing for a more thorough characterization of individuals presenting to EDs with OOD including demographics, comorbidities, OOD agent, and concepts that are often incompletely represented in ED notes such as the source of the opioid, intentionality of OOD, and ED treatment and discharge disposition. Our goal is to create tools and training materials that extend modifications from the initial set of institutions to the entire ACT network (60 or so CTSA institutions), so that the entire network can be used for trial planning and surveillance.

## MATERIALS AND METHODS

### Network infrastructure

**Figure 1.**
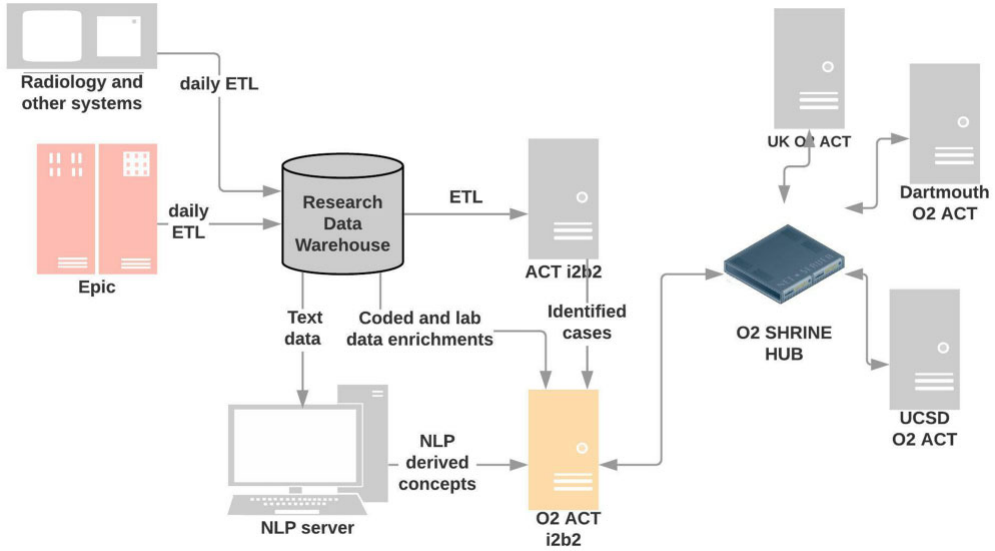
Overall architecture of O2-NET.

The O2-Network is a federated research network designed around a parallel implementation of i2b2 and SHRINE to existing data assets. We chose this approach to allow rapid testing and adaptation of the network for critical issues. The network was anticipated to be in a development phase for several years, with the eventual goal of merging innovations into production i2b2 and SHRINE environments for National Center for Advancing Translational Sciences (NCATS) through appropriate governance processes. Sites were required to clone and install a second ACT i2b2 instance and SHRINE node for this purpose. The design of the network is shown in Figure 1.

### E-phenotype definition

The ICD-10 and laboratory data-based e-phenotyping team was led by an OUD researcher. The primary objective of this team is to develop precise OOD cohort entry descriptions for surveillance databases. This team’s approach was focused on the harmonization of prior literature on definitions of OOD published in the literature. This team conducted a scoping review^14^ of the literature on ICD-driven phenotypes for identification of OOD using PubMed search with snowball sampling of the literature cited by discovered papers. Supplementary Appendix Table S1 summarizes these papers. The result suggested a tiered framework for the representation of the probability of a true case was necessary for complete data capture. High-probability cases would probably have an OOD-specific ICD-9 or ICD-10 code at the time of case abstraction or other coded evidence. Medium and low probability cases would be OOD cases that had some other medical problem that superseded the OD in retrospective coding and/or physician billing codes or had less convincing evidence in coded data. The specific ICD-10 codes used to classify cases as shown in Supplementary Appendix Table S2. To validate the framework, assess variability in coding, and support refinement of the e-phenotype using machine learning (ML) approaches, we are creating a gold standard data set. ED visit clinical notes classified into “at-risk” or OD events, based on an early ICD-10-based case definition, were exported into an REDCap application. Remote and in-person reviewers used this application to annotate notes with a document-level classification of the probability of the case representing an OOD event. Our aim is to review around 2250 cases, based on the training set size desired for ML and sample size calculations for the precision of estimates. A team of 12 reviewers (clinicians, clinical psychologists, and medical students) was assembled and is working collaboratively from multiple institutions using the REDCap application.

### Application of NLP, ML, and artificial intelligence methods

The primary goal of the NLP team is to enrich data in identified cases with critical concepts for OUD and OOD in the notes but which are unreliably coded. The NLP team was led by an experienced informatician and its objectives and work reflected that perspective. *Their approach was focused on abstraction at the sentence level of medical facts* about the case (as opposed to the other team’s work on the classification of cases for cohort generation. We initially envisioned 2 types of inferred information that might be abstracted from a note (1) documented response to OOD treatment (“Narcan” or “naloxone”) confirming the diagnosis of OD and (2) the “intentionality” of the OD. For example, “Was this a patient with chronic pain who accidentally used too much medication, an individual with OUD who unanticipatedly took a more concentrated aliquot of the drug, or was this a suicide attempt?” Such inferences would valuable information for clinical intervention development and trial planning.

Our initial work evaluated several different approaches to concept identification that combined ML and NLP methods using the CLAMP platform.^15^ Four NLP approaches were developed: (1) Named Entity Recognition (NER) + Rules, (2) A Support Vector Machine (SVM) classifier based on java libsvm library, with unigrams, bigrams, and trigrams as a feature; (3) A Neural Network approach based on pretrained context embeddings: Bidirectional Encoder Representations from Transformers (BERT) was used for the classification task, and (4) BERT+ Rules. Modifiers such as response to naloxone and intentionality were detected for 2 primary entities (opioid and naloxone). Inference rules were applied to further eliminate false positives, including situations such as the subject is not the OD patient, an OD event did not happen (eg, condition, hypothesis), or a negation (eg, “no,” “deny”). NLP performance was evaluated using the gold standard of manual review of potentially relevant sentences identified by Ruta.

### EHR modifications

While we were optimistic about the potential role of NLP and other tools in enhancing available data from patients previously treated for OOD, we believed it was preferable for incident cases to improve provider documentation of the OOD during clinical care. To achieve this goal, we worked with physicians and OUD experts to develop an enhanced template for documentation of provider notes in the ED so the necessary data for trial planning would be captured. The team was led by an ED clinician with a research focus on OUD. The goal was to produce a clinically useful tool to enhance data capture for the OOD clinical context.

**Figure 2.**
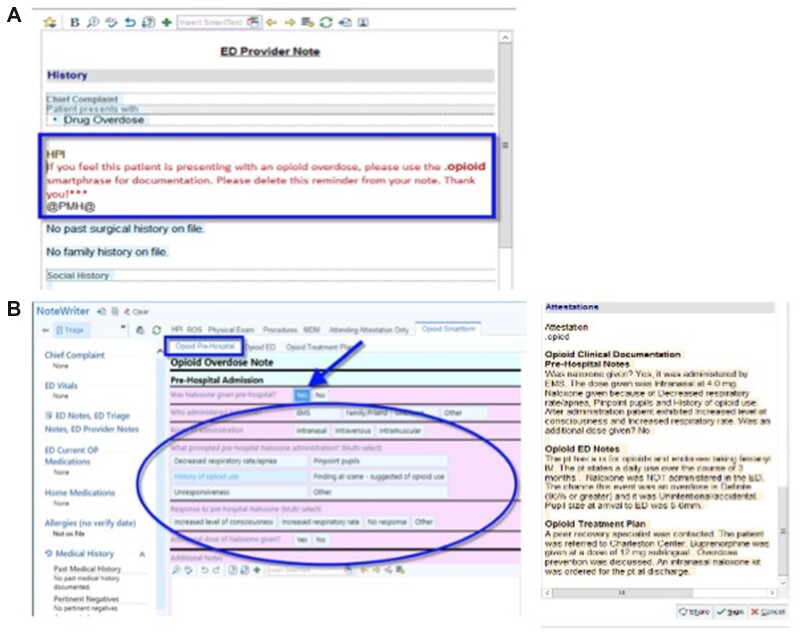
(A) Reminder notice inserted into emergency department notes as it appears in the electronic health record (Epic) based on a patient’s chief complaint. (B) OST for documenting an overdose episodes and resulting provider note. Screen images used with permission from the electronic health record vendor (Epic).

To make the additional documentation for the research database more palatable for providers, the OOD template or Opioid Smart Tool (OST) was designed to automatically produce a text note for the clinician by selecting from alternatives or filling in specific blanks. The OST is shown in Figure 2b. The OST also incorporated tools for changing physician behavior including a prompt to prescribe naloxone for home use, a recognized treatment that is widely underutilized.^16^^,^^17^ When during the initial deployment, we observed no clinical usage of the OST by providers, we added a reminder system, triggered by the patient’s chief complaint. If the words, “Drug Overdose,” “naloxone,” or “Narcan” were identified in the chief complaint. This reminder, rather than being a pop-up style alert, inserted a “reminder” phrase into the history section of the ED provider’s note suggesting that they use the documentation template. Figure 2a shows this reminder in context. One concern was whether this type of prompting would be acceptable to users. Therefore, we assessed usability and provider satisfaction among users of the prompt through the System Usability Scale (SUS) and Net Promoter Survey (NPS), through an anonymous email survey. The study of user acceptance, in the context of live deployment across the health system, was determined to be a quality improvement activity and as such, was exempt from human subjects review on that basis.

### Evaluation

As specified in the grant application, the evaluation of the impact of the network was to be based on the network’s value in generating viable research processes for ED-based OOD mitigation research. This was proposed to be studied in a series of annual retreats which combined discussions of developments in opioid abuse treatment in emergency settings with results from the network, to generate hypotheses and proposals for studies. Discussions were recorded and qualitative analytical methods were to be used to assess the impact of network data on hypothesis generation. The first evaluation conference was held (virtually) in November of 2021.

## RESULTS

Table 1 shows the progress of the O2 Network at the end of year 2. As shown in the table, there was substantial advancement not only with the development of OOD tools but significant work on implementation. All remote sites completed initial queries of coded e-phenotypes, deployed i2b2 and SHRINE instances, initiated deployment of NLP pipelines, and started to seek institutional governance approval of ED EHR template tools after identification of clinical champions and approval of installation by those champions.

**Table 1.** Progress on the creation of O2-Net across the consortium (counts rounded to the nearest 10 persons) based on ICD codes

	Near certain cases count (e-phenotypes 1 and 2)	Probable case count (e-phenotypes 3 and 4)	Possible case count (e-phenotype 5)	i2b2 for O2-Net	SHRINE	NLP pipeline	OST deployment
MUSC	12 230	3270	4080	Operational	Operational	Operational	Deployed
Dartmouth	1580	280	260	Operational	Operational	Deploying	Seeking IT governance approval
UK	15 250	18 140	14 060	Operational	Operational	Deploying	Seeking IT governance approval
UCSD	8800	6740	18 310	Operational	Operational	Deploying	Seeking IT governance approval

Table highlights differences in case make-up.

NLP: natural language processing.

### E-phenotype development

The proposed *a priori* case definitions for each category and associated ICD-10 codes resulting are presented in Supplementary Table S2. Case counts were obtained from each site between October 2015 and April 2020 (patients aged 12 and older) in which the identified ICD-10 codes had been used in any diagnostic section of the ED visit record. The percentage of “definite” cases seen at each institution ranged from 74.5% (UK) to 26% (UCSD) across the network, suggesting substantial variability in coding practices. Ongoing work is evaluating the accuracy and calibration of the mapped codes and any improvements in categorization afforded by ML and AI methods.

### NLP and ML/AI development

Tools combining AI and NLP approaches proved robust at identification and/or inference of targeted clinical concepts. NER+ Rules identified 1513 candidate opioid-related OD content sentences from a random 20% sample of 2.47 million clinical notes. The results demonstrated that the DL approach, BERT, with postprocessing rules achieved the best performance at inference with a precision of 0.97 and recall of 0.90 (F Score 0.94). NLP tools were then modified for installation using Docker and distributed to other sites in the consortium. Future work will examine the portability of these methods.

### EHR documentation tools

As shown in Table 2, the OST reminder triggered in roughly 21% of cases categorized as high probability (*n* = 81) using postdischarge coded data during the test period at MUSC Charleston. The MUSC hospital system had recently acquired 4 regional community hospitals, and we were surprised to see that ED providers at the newly acquired regional hospitals were also using the OST, despite not having received any training. In a subset of cases seen in these facilities (*n* = 130), the rate of the template used in high-probability cases was 14%. Among respondents (*n*-12), the overall SUS score was 72.5% and the NPS score was 74.2%.

**Table 2.** Opioid smart tool “reminder” trigger and use counts (percentages) in high-probability cases at MUSC Charleston and within the MUSC regional hospitals

Location	MUSC Charleston	MUSC regional hospitals
High-probability cases	389	292
Template triggered by real-time data	81 (21%)	130 (45%)
Template used for documentation	23 (28%)	18 (14%)

While not a primary goal of this study, we noted that the OST was highly effective in changing provider behavior. In cases where the template was used, the prescription of naloxone for home use was much higher than when it was not used. At MUSC, when the template was used, home naloxone was prescribed 66% of cases versus 16% when it was not used (*P* < .0001, Fisher’s exact test). At community hospitals, the use of the template was concomitant with naloxone prescription in 44% of cases, versus in 16% of cases when the template was not used (*P* < .0001, Fisher’s exact test).

### Evaluation

The evaluation retreat was held virtually using Zoom conferencing^18^ due to the coronavirus disease 2019 (COVID-19) pandemic. It included both scientific presentations, network updates, and discussions of potential uses of the network. While detailed analyses of qualitative data from discussions are ongoing, opioid researchers saw the initial results on the use of the OST on provider behavior for naloxone for home use prescriptions as encouraging and suggested the development of a clinical effectiveness study based on deployment of this technology. Informatics researchers saw additional value in the randomization of providers to different informatics methods for prompting reminders to prescribe. One arm might use a more traditional approach of pop-up style alerts, which has been previously tried in clinical practice with variable success.^19^^,^^20^ The other might use the OST with its reminder integrated into the physician’s note. A trial-based comparison of these different modes for delivery of decision support is under development. Researchers at Dartmouth and UCSD sites are implementing the OST to determine if results seen at MUSC on the prescription of Naloxone for home use are replicated at their institutions.

## DISCUSSION

Investments in research infrastructure need to be adaptable to the evolving demands of society for health research. In this paper, we describe our experiences in the first 2 years of operations, adapting the ACT network to a new use case: surveillance of, and planning for, trials to address the opioid epidemic. The approach has 3 areas of emphasis that are likely to be important elements of any focused registry-like activity: (1) e-phenotype definition and harmonization of coding, (2) enrichment of extent data with NLP of clinical notes, and (3) enhancement of prospective capture of data with informatics-based tools. Each of these areas posed unique challenges.

Persistent challenges include variability in coding^21^^,^^22^ and potential differences in the sensitivity and specificity of e-phenotype definitions across institutions sharing a common data model.^23^ However, substantial progress was made in deploying NLP tools across the network and in the adoption of the OST template.

The question of whether a general-purpose federated research network such as ACT can be easily extended, at a low cost, to address targeted and emergent problems remains an open one. At the minimum, long-term support for a network needs to be assured, which is not the case for the ACT network at this time. NCATS has prioritized the centralization of data resources through its work on COVID-19 over federated networks.^24^

There are many challenges in adapting a federated network to a new task, and, as often is true in informatics, the technical challenges are the least significant ones. However, it is clear no single technology in isolation (e-phenotyping, NLP, or documentation templates) can solve all the data issues necessary for a research network. Improved primary data collection, while difficult, through tools such as the OST, may be the only viable option at times. Furthermore, even with the completion of a network, its use for the acceleration of research is hard to ensure. In this case, a fortuitous finding related to the use of the OST, amplified by an informed interdisciplinary discussion led to a viable hypothesis for implementation research. The process of how new data triggers research hypotheses deserves further study. In the movement from hypothesis to study, a federated network may have certain advantages. A federated model respects each sites unique clinical processes and equips each site with resources that allows replication of early findings, as is being done with the OST from this study, potentially accelerating translation.

### Conclusions

Our initial work suggests it is feasible and valuable to extend the ACT network infrastructure, with its real-time query capabilities, to begin addressing the challenges of responding to the opioid pandemic in a federated model.

## FUNDING

This publication [or project] was supported, in part, by the National Center for Advancing Translational Sciences of the National Institutes of Health under Grant Numbers U01TR002628, UL1TR001450, UL1TR001998, UL1TR001442, and UL1TR001086. The content is solely the responsibility of the authors and does not necessarily represent the official views of the National Institutes of Health.

## AUTHOR CONTRIBUTIONS

Study design: LAL, VZ, LJ, JLM, JSO, SH, MH, and JCT. Acquisition, analysis, and interpretation of data: VZ, LJ, JSO, RW, LAM, PS, and DRH. Drafting of the manuscript and revision of content: all authors. Final approval of the manuscript and agreement to be accountable for all aspects of the work and resolution of all questions related to accuracy or integrity of the manuscript: all authors.

## Supplementary Material

ooac055_Supplementary_Data
