# Supplementary material for: Enhancing research data infrastructure to address the opioid epidemic: the Opioid Overdose Network (O2-Net)
Source: JAMIA Open. 2022 Jun 30;5(2):ooac055. doi: 10.1093/jamiaopen/ooac055 (PMC9243402; doi:10.1093/jamiaopen/ooac055)
Supplement: ooac055_Supplementary_Data [file ooac055_supplementary_data.zip › Supplemental Table 2.docx]

**Supplemental Table 2.** Case definitions and ICD-10 codes for five e-phenotype categories.

# Categorization Definition ICD-10 Codes

**Definite via Administrative Coding**

(PPV≧95%)

# Poisoning or adverse effect of opioid (narcotic), initial encounter or sequelae

T40.0 T40.1 T40.1X2A T40.2 T40.2X2A

T40.2X5A T40.2X5S T40.3 T40.3X5A T40.4 T40.6 T40.601A T40.601S T40.602A T40.603S T40.604A T40.605A T40.691A T40.695A T400X1A T400X1S T400X2A T400X2S T400X3A T400X3S T400X4A T400X4S T400X5A T400X5S T401X1A T401X1S T401X2S T401X3A T401X3S T401X4A T401X4S T401X5A T401X5S T402X1A T402X1S T402X2S T402X3A T402X3S T402X4A T402X4S T403X1A T403X1S T403X2A T403X2S T403X3A T403X3S T403X4A

# Definite via Naloxone Administration

(PPV≧95%)

# CNS Depression/Altered Mental Status/Poisoning or adverse effect of unspecified substance

T403X4S T403X5S T404X1A T404X1S T404X2A T404X2S T404X3A T404X3S T404X4A T404X4S T404X5A T404X5S T40602S T40603A T40603S T40604S T40605S T40691S T40692A T40692S T40693A T40693S T40694A T40694S T40695S X42

X62 Y12

T4391XS T4392XA T4392XS T4393XA T4393XS T4394XS T6591XS T6592XA T6592XS T6593XA

T6593XS T6594XS J80 J9600 J9601 J9602 J9690 J9691 J9692 R400 R401 R4020 R402110 R402111 R402112

|  | R402113 | |
| --- | --- | --- |
|  | R402114 | |
|  | R402120 | |
|  | R402121 | |
|  | R402122 | |
|  | R402123 | |
|  | R402124 | |
|  | R402210 | |
|  | R402211 | |
|  | R402212 | |
|  | R402213 | |
|  | R402214 | |
|  | R402220 | |
|  | R402221 | |
|  | R402222 | |
|  | R402223 | |
|  | R402224 | |
|  | R402310 | |
|  | R402311 | |
|  | R402312 | |
|  | R402313 | |
|  | R402314 | |
|  | R402320 | |
|  | R402321 | |
|  | R402322 | |
|  | R402323 | |
|  | R402324 | |
|  | R402340 | |
|  | R402341 | |
|  | R402342 | |
|  | R402343 | |
|  | R402344 | |
|  | R403 | |
|  | R404 | |
|  | R410 | |
|  | R4182 | |
|  | **AND: Administration of Naloxone** | None |
|  | **AND: Positive response to Naloxone** None | |
|  | **administration (observed or reported)** | |
|  | **NOT*:* Condition Responsive to** | Under Dev. |
|  | **Naloxone Administration but not** |  |
|  | **opioid overdose** |  |
| **Probable via Naloxone Administration CNS Depression/Altered Mental** T4391XS (PPV ≧50% and <95%) **Status/Poisoning or adverse effect as** T4392XA  **a result of an unspecified substance** T4392XS  T4393XA T4393XS T4394XS T6591XS | | |

T6592XA T6592XS

T6593XA

T6593XS

T6594XS J80 J9600 J9601 J9602 J9690 J9691 J9692 R400 R401 R4020 R402110 R402111 R402112 R402113 R402114 R402120 R402121 R402122 R402123 R402124 R402210 R402211 R402212 R402213 R402214 R402220 R402221 R402222 R402223 R402224 R402310 R402311 R402312 R402313 R402314 R402320 R402321 R402322 R402323 R402324 R402340 R402341 R402342 R402343 R402344 R403 R404 R410

|  | R4182 | |
| --- | --- | --- |
|  | **AND: Evidence of opioid abuse or** | F11.1 |
|  | **dependence** |  |
|  |  | F11.2 |
|  |  | F11.9 |
|  |  | F11.93 |
|  |  | F11.94 |
|  |  | F11.988 |
|  |  | F1110 |
|  |  | F11120 |
|  |  | F11121 |
|  |  | F11122 |
|  |  | F11129 |
|  |  | F1114 |
|  |  | F11150 |
|  |  | F11151 |
|  |  | F11159 |
|  |  | F11181 |
|  |  | F11182 |
|  |  | F11188 |
|  |  | F1119 |
|  |  | F1120 |
|  |  | F1121 |
|  |  | F11220 |
|  |  | F11221 |
|  |  | F11222 |
|  |  | F11229 |
|  |  | F1123 |
|  |  | F1124 |
|  |  | F11250 |
|  |  | F11251 |
|  |  | F11259 |
|  |  | F11281 |
|  |  | F11282 |
|  |  | F11288 |
|  |  | F1129 |
|  |  | F1190 |
|  |  | F11921 |
|  |  | F11922 |
|  |  | F11950 |
|  |  | F11951 |
|  |  | F11959 |
|  |  | F11981 |
|  |  | F11982 |
|  |  | R826 |
|  |  | R892 |
|  |  | R893 |
|  | **NOT: Poisoning or adverse effect of** | N/A |
|  | **identified non-opioid substance** |  |
| **Probable via Other**  (PPV ≧50% and <95%) | **CNS Depression/Altered Mental Status/Poisoning or adverse effect of unspecified substance** | T4391XS T4392XA T4392XS |

T4393XA T4393XS T4394XS T6591XS T6592XA T6592XS T6593XA T6593XS T6594XS J80 J9600 J9601 J9602 J9690 J9691 J9692 R400 R401 R4020 R402110 R402111 R402112 R402113 R402114 R402120 R402121 R402122 R402123 R402124 R402210 R402211 R402212 R402213 R402214 R402220 R402221 R402222 R402223 R402224 R402310 R402311 R402312 R402313 R402314 R402320 R402321 R402322 R402323 R402324 R402340 R402341 R402342 R402343 R402344

|  | | R403 | |
| --- | --- | --- | --- |
|  |  | R404 | |
|  |  | R410 | |
|  |  | R4182 | |
|  |  | **AND: Administration of Naloxone** | None |
|  |  | ***Without:*** *Assessment of response to Naloxone* | None |
|  |  | **AND: Positive Tox Screen for Opioid/** R826  **Narcotic** R892 | |
|  |  | R893  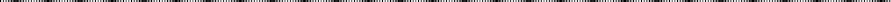 | |
|  |  | ***OR:*** *Current (Past 3 Months)*  *Prescription for Opioid/ Narcotic* | None |
| **Possible** |  | **CNS Depression/Altered Mental** | J80 |
|  | (PPV ≧5% and <50%) | **Status/Poisoning or adverse effect of** | J9600 |
|  |  | **any substance** | J9601 |
|  |  |  | J9602 |
|  |  |  | J9690 |
|  |  |  | J9691 |
|  |  |  | J9692 |
|  |  |  | R400 |
|  |  |  | R401 |
|  |  |  | R4020 |
|  |  |  | R402110 |
|  |  |  | R402111 |
|  |  |  | R402112 |
|  |  |  | R402113 |
|  |  |  | R402114 |
|  |  |  | R402120 |
|  |  |  | R402121 |
|  |  |  | R402122 |
|  |  |  | R402123 |
|  |  |  | R402124 |
|  |  |  | R402210 |
|  |  |  | R402211 |
|  |  |  | R402212 |
|  |  |  | R402213 |
|  |  |  | R402214 |
|  |  |  | R402220 |
|  |  |  | R402221 |
|  |  |  | R402222 |
|  |  |  | R402223 |
|  |  |  | R402224 |
|  |  |  | R402310 |
|  |  |  | R402311 |
|  |  |  | R402312 |
|  |  |  | R402313 |
|  |  |  | R402314 |
|  |  |  | R402320 |
|  |  |  | R402321 |
|  |  |  | R402322 |


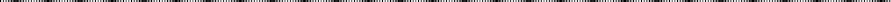


R402323 R402324 R402340 R402341 R402342 R402343 R402344 R403 R404 R410 R4182 T398X2S T398X3A T398X3S T398X4A T398X4S T3991XS T3992XA T3992XS T3993XA T3993XS T3994XA T3994XS T405X1S T405X2A T405X2S T405X3A T405X3S T405X4S T407X1S T407X2A T407X2S T407X3A T407X3S T407X4S T408X1S T408X2A T408X2S T408X3A T408X3S T408X4S T40901S T40902A T40902S T40903A T40903S T40904S T40991S T40992A T40992S T40993A T40993S T40994S T423X1S

T423X2A T423X2S T423X3A T423X3S T423X4S T424X1S T424X2A T424X2S T424X3A T424X3S T424X4S T426X1S T426X2A T426X2S T426X3A T426X3S T426X4A T426X4S T4271XS T4272XA T4272XS T4273XA T4273XS T4274XS T43601S T43602A T43602S T43603A T43603S T43604S T43621S T43622A T43622S T43623A T43623S T43624S T43631S T43632A T43632S T43633A T43633S T43634S T43691S T43692A T43692S T43693A T43693S T43694S T438X1S T438X2A T438X2S T438X3A T438X3S T438X4S

T4391XS T4392XA T4392XS T4393XA T4393XS T4394XS T481X1S T481X2A T481X2S T481X3A T481X3S T481X4S T48201S T48202A T48202S T48203A T48203S T48204S T48291S T48292A T48292S T48293A T48293S T48294S T483X1S T483X2A T483X2S T483X3A T483X3S T483X4S T50901A T50901S T50902A T50902S T50903A T50903S T50904A T50904S T50991S T50992A T50992S T50993A T50993S T50994S T510X1S T510X2A T510X2S T510X3A T510X3S T510X4S T511X1S T511X2A T511X2S T511X3A

|  | T511X3S | |
| --- | --- | --- |
|  | T5191XS | |
|  | T5192XA | |
|  | T5192XS | |
|  | T5193XA | |
|  | T5193XS | |
|  | T5194XS | |
|  | T520X1S | |
|  | T520X2A | |
|  | T65211S | |
|  | T65212A | |
|  | T65212S | |
|  | T65213A | |
|  | T65213S | |
|  | T65214S | |
|  | T65221S | |
|  | T65222A | |
|  | T65222S | |
|  | T65223A | |
|  | T65223S | |
|  | T65224S | |
|  | T65291S | |
|  | T65292A | |
|  | T65292S | |
|  | T65293A | |
|  | T65293S | |
|  | T65294S | |
|  | T65891S | |
|  | T65892A | |
|  | T65892S | |
|  | T65893A | |
|  | T65893S | |
|  | T65894S | |
|  | T6591XS | |
|  | T6592XA | |
|  | T6592XS | |
|  | T6593XA | |
|  | T6593XS | |
|  | T6594XS | |
|  | **AND: Positive Tox screen for Opioid/** R826  **Narcotic**  R892  R893  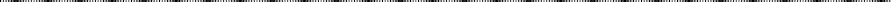 | |
|  | ***OR:*** *Current (Past 3 Months)* | None |
|  | *Prescription for Opioid/ Narcotic* |  |
