# Supplementary material for: Enhancing research data infrastructure to address the opioid epidemic: the Opioid Overdose Network (O2-Net)
Source: JAMIA Open. 2022 Jun 30;5(2):ooac055. doi: 10.1093/jamiaopen/ooac055 (PMC9243402; doi:10.1093/jamiaopen/ooac055)
Supplement: ooac055_Supplementary_Data [file ooac055_supplementary_data.zip › Supplemental_Table_1.docx]

| **First Author** | **Year** | **Country** | **Title** | **ICD Codes** |
| --- | --- | --- | --- | --- |
| Badger | 2019 | US | Machine learning for phenotyping  opioid overdose events. | None Provided |
| Binswanger | 2019 | US | Development and evaluation of a  standardized research definition for opioid overdose outcomes. | None Provided |
| Campbell | 2018 | US | Use of a prescription opioid  registry to examine opioid misuse and overdose in an integrated health system. | ICD-9 (9650, 9650.0, 9650.1, 9650.2,  9650.9, E850.0, E850.1, E850.2) |
|  |  |  |  | ICD-10 (T40.0, T40.1, T40.2, T40.3, T40.4, X42, X62, Y12) |
| Coben | 2010 | US | Hospitalizations for poisoning by  prescription opioids, sedatives, and tranquilizers. | ICD-9 (960-979; specifically: 965.01,  965.02, 965.09) |
| Daly | 2017 | US | Use of Emergency Department  Data to Monitor and Respond to an Increase in Opioid Overdoses in New Hampshire, 2011-2015. | iCD-10 (F11.1, F11.2,F11.9, F19.2,  T40.0x excluding T40.0X6, T40.1X, T40.3X excluding T40.3X6, T40.2X excluding T40.2X6, T40.6 excluding T40.606 and T40.696) |
| Dunn | 2010 | US | Opioid prescriptions for chronic  pain and overdose: a cohort study. | ICD-9 (9650*, E850.1, E950.0, E980.0)  and ICD-10 (T40.0, T40.2, T40.3, T40.4, X42, X62, Y12) |
|  |  |  |  | ICD-9 (E935.0, E935.1, E935.2) and ICD-10 (Y45.0) |
|  |  |  |  | ICD-9 (276.4, 292.1, 292.81, 292.8*,  486, 496, 518.81, 518.82, 780.0,  780.97, 786.03, 786.05, 786.09,  786.52, 799.0*, E950-959 (n/a,  suicide)) |

**Supplemental Table 1.** Studies referenced in literature review, including ICD codes.

| Ellison | 2016 | US | Identifying Patients for Overdose  Prevention With ICD-9 Classification in the Emergency Department, Massachusetts, 2013-  2014. | None Provided |
| --- | --- | --- | --- | --- |
| Green | 2017 | US | Assessing the accuracy of opioid  overdose and poisoning codes in diagnostic information from electronic health records, claims data, and death records. | ICD-9 (965.00, 965.01, 965.02, 965.09,  E850.0. E850.1, E850.2) |
|  |  |  |  | ICD-10 (T40.0, T40.1, T40.2, T40.3, T40.4, X42, X62, Y12) |
|  |  |  |  | ICD-9 (E935.0, E935.1, E935.2, Y45) |
|  |  |  |  | ICD-9 (276.4, 292.1, 292.81, 292.8,  486, 496, 518.81, 518.82, 728.88,  780.0, 780.97, 786.03, 786.05, 786.09,  786.52, 799.0, 970.1, E950-E959, E962) |
| Green | 2019 | US | Identifying and classifying opioid-  related overdoses: A validation study. | ICD-9 (965.00; 965.01; 965.02; 965.09;  E850.0; E850.1; E850.2; 9650; T40.0; T40.1; T40.2; T40.3; T40.4; X42; X62; Y12) |
|  |  |  |  | ICD-9 ( E950.0; E965.01; E850.0;  T40.1) |
|  |  |  |  | ICD-9 (E950.0; E950.1; E950.2; E950.3; E950.4; E950.5; E950.9; E956; E958.8; E958.9; V62.84) |
|  |  |  |  | ICD-9 (291.x, 303.x, 305.0; 300.0,  300.2, 300.3, 309.20, 309.21, 309.24,  309.91; 296.0, 296.1, 296.4-296.7,  296.8, 296.81, 296.89; 292.x, 304.x,  305.2-305.9; 297.1, 297.3, 298.8,  298.9, 301.22; 295.x; 305.1, 649.0,  989.84, V15.82) |
|  |  |  |  | ICD-9 (965.01, E850.0, T40.1, 965.00,  965.02, 095.09, 304.00-304.03, 304.70-  304.72, 305.50-305.53, 292.x, 291.x,  303.x, 305.0, 305.1, 649.0, 989.84, V15.82) |
| Green | 2019 | US | Development of an algorithm to  identify inpatient opioid-related overdoses and oversedation using | None Provided |
| Guy | 2018 | US | Emergency Department Visits  Involving Opioid Overdoses, U.S., 2010-2014. | ICD-9 (965.00, 965.02, 965.09, E850.1,  E850.2, 965.01, E850.0) |

| Hasegawa | 2014 | US | Trends in U.S. emergency  department visits for opioid | ICD-9 (965.0X) |
| --- | --- | --- | --- | --- |
|  |  |  | overdose, 1993-2010. |  |
| Hazlehurst | 2019 | US | Using natural language processing  of clinical text to enhance | None Provided |
|  |  |  | identification of opioid-related |  |
|  |  |  | overdoses in electronic health |  |
|  |  |  | records data. |  |
| Hedegaard | 2017 | US | Proposed ICD-10-CM Surveillance | ICD-9 (T36-T50 with a 6th character of |
|  |  |  | Case Definitions for Injury | 1,2,3, or 4 (Exceptions: T36.9, T37.9, |
|  |  |  | Hospitalizations and Emergency | T39.9, T41.4, T42.7, T43.9, T45.9, |
|  |  |  | Department Visits. | T47.9, and T49.9 with a 5th character |
|  |  |  |  | of 1, 2, 3, or 4)) |
|  |  |  |  | ICD-9 (T51-T65) |
| Hsu | 2017 | US | Hospitalizations, costs and | ICD-9 (965.01, E850.0, 965.00, 965.02, |
|  |  |  | outcomes associated with heroin | 965.09, E850.01, E850.02) |
|  |  |  | and prescription opioid overdoses |  |
|  |  |  | in the United States 2001-12. |  |
| Larochelle | 2016 | US | Opioid Prescribing After Nonfatal | ICD-9 (965.00, 965.02, 965.09, E850.1, |
|  |  |  | Overdose and Association With | E850.2, 965.01, E850.0) |
|  |  |  | Repeated Overdose. |  |
| Lovegrove | 2019 | US | US Emergency Department Visits | None Provided |
|  |  |  | for Acute Harms From |  |
|  |  |  | Prescription Opioid Use, 2016- |  |
|  |  |  | 2017. |  |
| Maeng | 2017 | US | Patterns of health care utilization  and cost before and after opioid overdose: findings from 10-year longitudinal health plan claims | ICD-9 (965.00, 965.01, 965.02, 965.09  or E850.0, E850.1, E850.2, E935.0, E935.1, E935.2) |
|  |  |  | data. |  |
| Mosher | 2017 | US | Trends in Hospitalization for | ICD-9 (965.00, 965.01, 965.09, E850.0, |
|  |  |  | Opioid Overdose among Rural | E850.2; E935.0 was exclusionary for |
|  |  |  | Compared to Urban Residents of | POD indicating adverse effects of |
|  |  |  | the United States, 2007-2014. | therapeutic heroin) |
| Mountcastle | 2019 | US | Validation of an administrative | ICD-9 ((965.00, 965,02, 965.09, |

claims coding algorithm for serious E850.1, E850.2, E935.1, and E935.2) opioid overdose: A medical chart

review.

ICD-9 (518.81, 786.03, 799.01, 799.02,

799.1, 780.0, 780.01, 780.02, 780.09)

CPT (31500, 94002, 94660, and

99291)

| Nolan | 2017 | US | Developing Syndromic  Surveillance to Monitor and Respond to Adverse Health Events Related to Psychoactive Substance Use: Methods and Applications. | None Provided |
| --- | --- | --- | --- | --- |
| Olfson | 2018 | US | Service Use Preceding Opioid-  Related Fatality. | ICD-9 (965.0, E850.0, E850.1, E850.2) |

|  |  |  |  | ICD-10 (T40.0, T40.1, T40.2, T40.3,  T40.4, T40.6, T42.4) |
| --- | --- | --- | --- | --- |
| Rowe | 2017 | US | Performance Measures of  Diagnostic Codes for Detecting Opioid Overdose in the Emergency Department. | ICD-9 (E850.0-2; E965.0-9) |
|  |  |  |  | ICD-9 (977.9; E858.9, E980.0) |
|  |  |  |  | ICD-9 (965) |
|  |  |  |  | ICD-9 (305.50-305.52) |
|  |  |  |  | ICD-9 (305.90-305.93) |
|  |  |  |  | ICD-9 (305) |
|  |  |  |  | ICD-9 (780.97; V71.89; 780.2; E980.3;  V62.84; 304; V71.9) |
|  |  |  |  | ICD-9 (333.9; 578.1; 303; 780.79;  276.51) |
| Salzman | 2019 | US | Epidemiology of opioid-related  visits to US Emergency Departments, 1999-2013: A retrospective study from the NHAMCS (National Hospital Ambulatory Medical Care Survey). | ICD-9 (305.5X; 304.0X; 965.0X;  304.7X) |
| Slavova | 2014 | US | Drug overdose surveillance using  hospital discharge data. |  |
| Unick | 2013 | US | Intertwined epidemics: national  demographic trends in hospitalizations for heroin- and opioid-related overdoses, 1993-  2009. | ICD-9 (965.00, 965.01, 965.02, or  965.09) |
|  |  |  |  | ICD-9 (E850.0, E850.1, or E850.2) |
| Vivolo-Kantor | 2019 | US | Suspected Heroin Overdoses in  US Emergency Departments, 2017-2018. | ICD-9 (965.01, E850.0,  ICD-10 (T40.1X1A, T40.1X4A) |
| Youssef | 2018 | US | Characteristics of prior emergency  departments visits associated with subsequent opioid overdose. |  |
|  |  |  |  | ICD-9 (292.81) |
|  |  |  |  | ICD-9 (292.89) |
|  |  |  |  | ICD-9 (292.9) |
|  |  |  |  | ICD-9 (292.11) |
|  |  |  |  | ICD-9 (292.12) |
|  |  |  |  | ICD-9 (304) |
|  |  |  |  | ICD-9 (304.8) |
|  |  |  |  | ICD-9 (304.9) |
|  |  |  |  | ICD-9 (305.5) |
|  |  |  |  | ICD-9 (305.51) |
|  |  |  |  | ICD-9 (305.52) |

| ICD-9 (305.9) |
| --- |
| ICD-9 (796) |
| ICD-9 (965.09) |
| ICD-9 (977.9) |
| ICD-9 (989.9) |
